# Visual Corticocortical Inputs to Ferret Area 18

**DOI:** 10.3389/fnana.2020.581478

**Published:** 2020-10-06

**Authors:** Reem Khalil, Moody Roberne Jensy Saint Louis, Shaima Alsuwaidi, Jonathan B. Levitt

**Affiliations:** ^1^Department of Biology, Chemistry and Environmental Sciences, American University of Sharjah, Sharjah, United Arab Emirates; ^2^Department of Biology, City College of New York, New York, NY, United States; ^3^The Neuro, Montreal Neurological Institute-Hospital, McGill University, Montreal, QC, Canada; ^4^Graduate Center of the City University of New York, New York, NY, United States

**Keywords:** visual cortex, interareal projections, extrastriate, brain, V2, feedback

## Abstract

Visual cortical areas in the adult mammalian brain are linked by a network of interareal feedforward and feedback circuits. We investigated the topography of feedback projections to ferret (*Mustela putorius furo*) area 18 from extrastriate areas 19, 21, and Ssy. Our objective was to characterize the anatomical organization of the extrastriate feedback pool to area 18. We also wished to determine if feedback projections to area 18 share similar features as feedback projections to area 17. We injected the tracer cholera toxin B subunit (CTb) into area 18 of adult ferrets to visualize the distribution and pattern of retrogradely labeled cells in extrastriate cortex. We find several similarities to the feedback projection to area 17: (i) Multiple visual cortical areas provide feedback to area 18: areas 19, 21, Ssy, and weaker inputs from posterior parietal and lateral temporal visual areas. Within each area a greater proportion of feedback projections arises from the infragranular than from the supragranular layers. (ii) The cortical area immediately rostral to area 18 provides the greatest proportion of total cortical feedback, and has the greatest peak density of cells providing feedback to area 18. (iii) The spacing (peak cell density and nearest neighbor distances) of cells in extrastriate cortex providing feedback to areas 17 and 18 are similar. However, peak density of feedback cells to area 18 is comparable in the supra- and infragranular layers, whereas peak density of feedback cells to area 17 is higher in the infragranular layers. Another prominent difference is that dorsal area 18 receives a cortical input that area 17 does not: from ventral cortex representing the upper visual field; this appears to be roughly 25% of the feedback input to area 18. Lastly, area 17 receives a greater proportion of cortical feedback from area 21 than from Ssy, whereas area 18 receives more feedback from Ssy than from area 21. While the organization of feedback projections from extrastriate cortex to areas 17 and 18 is broadly similar, the main difference in input topography might arise due to differences in visual field representations of the two areas.

## Introduction

Mammalian visual cortical areas are linked via a system of feedforward (FF) and feedback (FB) circuits, which are thought to mediate different visual functions. FF projecting cells are found predominately in the supragranular layers while FB projecting cells are found largely in the infragranular layers (Rockland and Pandya, 1979; Kennedy and Bullier, 1985; Felleman and Van Essen, 1991; Payne and Lomber, 2003; Cantone et al., 2005). Studies in primates (Kennedy and Bullier, 1985; Felleman and Van Essen, 1991), cats (Batardiere et al., 1998), and rodents (Coogan and Burkhalter, 1990), also show that the laminar pattern of FF and FB projecting cells can be informative of hierarchical organization. FB circuits in the mammalian visual cortex are an important feature of cortical architecture. Others have suggested an important role for FB connections in mediating such functions as spatial integration (Levitt and Lund, 1997; Angelucci et al., 2002; Cantone et al., 2005), feature binding (Shipp et al., 2009), perceptual pop-out (Knierim and van Essen, 1992; Kastner et al., 1997), figure-ground segregation (Field et al., 1993; Kapadia et al., 1995; Lamme, 1995; Hupé et al., 1998; Bullier et al., 2001; Hupé et al., 2001), and attention (Huang et al., 2004). Prior studies have largely focused on the nature of FB inputs to area 17/V1 in adult primates (Rockland and Pandya, 1979; Zeki and Shipp, 1988), cats (Grant and Shipp, 1991; Batardiere et al., 1998), rodents (Coogan and Burkhalter, 1990; Yang et al., 2013), and ferrets (Cantone et al., 2005; Dell et al., 2019), yet detailed quantitative investigations focused on the organization of FB projections to area 18/V2 are scarce.

Anatomical work has been largely devoted to describing the overall areal and laminar pattern of FB to area 18/V2. Prior studies in primates have reported FB projections to area 18 (V2) from areas 19 (V3/anterior bank of the lunate sulcus (Tigges et al., 1981; Kennedy and Bullier, 1985), V4 (or prelunate gyrus) (Rockland and Pandya, 1979; Kennedy and Bullier, 1985; Nakamura et al., 1993; Nascimento-Silva et al., 2014), V5/MT (or superior temporal sulcus) (Maunsell and Van Essen, 1983; Kennedy and Bullier, 1985; Ungerleider and Desimone, 1986; Rockland and Knutson, 2000), and parietal association cortex (Borra and Rockland, 2011). Similarly, reports in cats have revealed FB projections to area 18 from areas 19 (Payne and Lomber, 2003), PMLS/AMLS/PLLS (Symonds and Rosenquist, 1984; Segraves and Innocenti, 1985), area 7 (Yang et al., 2016), and areas 20/21 (Bullier et al., 1984). Lastly, others have shown there are FB projections to ferret area 18 from areas 19, 21, Ssy, (Dell et al., 2019), 20a/20b (Dell et al., 2019), and PPr and PPc (Dell et al., 2019). Given that FB circuits are a ubiquitous feature with a critical role in visual cortical processing, it is important to document the pattern of FB to visual areas beyond V1.

A central goal of the present study was to describe and quantitatively assess the anatomical organization of FB connections to ferret area 18 from multiple extrastriate visual areas. We also wished to determine similarities and differences between cortical FB projections to area 18 with those to area 17. Lastly, we wanted to describe the laminar projection pattern of feedback connections to ferret area 18 in order to identify features of corticocortical connections that may be conserved across different mammalian species. We find that similar to area 17, the area immediately rostral to area 18 (area 19) provides the greatest proportion of cortical FB, followed by Ssy and area 21. Also similar to FB to area 17, within each cortical area providing FB to area 18 there is a greater proportion of FB projections arising from the infragranular than from the supragranular layers. Our results also reveal important differences in the topographic organization of FB projections that target areas 17 and 18 of the ferret visual cortex. Therefore, although many aspects of feedback circuitry to areas 17 and 18 are similar, there are also organizational differences that likely contribute to each area’s unique functional role. Understanding the nature of FB inputs to area 18 will illuminate the role of top–down processing in visual perception.

## Materials and Methods

### Anatomical Tracer Injections

We studied eight adult female sable ferrets (*Mustela putorius furo*; 0.8–1.2 kg). We used 6 ferrets for area 18 injections and 2 ferrets for area 17 injections. One of the area 18 cases intruded slightly onto area 17 and was thus used to compare with the other cases. Animals were obtained from Marshall Farms (North Rose, NY, United States) and housed under a 12-h light/dark cycle. All procedures conformed to National Institutes of Health guidelines. The complete protocol used for anatomical tracer injections using Cholera toxin B subunit (CTb) was previously described (Khalil and Levitt, 2014; Khalil et al., 2018). Briefly, animals were sedated prior to surgery with an intramuscular injection of ketamine (25 mg/kg) and xylazine (2 mg/kg), and their heads were fixed with a stereotaxic apparatus and secured with ear bars. A mask was placed on the nose and snout to administer isoflurane throughout the surgery. The animals were respired using a pump, which delivered a mixture of 1–2% isoflurane, in O_2_. The EKG, pulse, tissue oxygenation, and rectal temperature were continuously monitored throughout the surgery, and maintained at appropriate levels. During a sterile surgery, Lidocaine HCl was injected into the scalp prior to incisions. The scalp was retracted, and a craniotomy and durotomy were performed on either the left or right hemisphere. Cholera toxin B subunit (CTb: List Biological Laboratories, Campbell, CA, United States, Cat. no. 104) was reconstituted in 0.1 M potassium phosphate buffer (PB, 1%, pH 6.0) and either pressure-injected or delivered with current into area 18 (approximately 2.3 mm from the occipital pole). Iontophoretic injections using glass micropipettes (aperture 10–15 μm) were made by passing current at 2 μA for 10 min with a 7-s on–off cycle at two cortical depths to ensure that the extent of the injection site spanned both the upper and lower layers of the cortex. Alternatively, pressure injections were delivered with a Picospritzer (Parker Hannifin, Fairfield, NJ, United States), using glass micropipettes (aperture 30–40 μm) at two cortical depths with 2 × 10 ms pulses at each location. The average injection core diameter was ∼1200 μm. Both injection methods yielded comparable injection core volumes. Animals were given postoperative antibiotic (ampicillin: 25 mg/kg) and analgesic (buprenorphine: 0.05 mg/kg) for 2 days. After a survival period of 7–10 days, the animals were deeply anesthetized with ketamine (25 mg/kg) + xylazine (2 mg/kg), given an intraperitoneal dose of 15 mg/kg of sodium selenite for subsequent labeling of synaptic zinc, then 45–60 min later were euthanized with an intraperitoneal overdose of pentobarbital (100 mg/kg).

### Tissue Processing and Anatomical Delineation of Visual Areas

The complete protocol for tissue fixation and CTb immunohistochemistry is described in detail elsewhere (Khalil and Levitt, 2014; Khalil et al., 2018). Briefly, animals were transcardially perfused using saline solution followed by a 4% paraformaldehyde solution, then a 4% paraformaldehyde plus 10% sucrose solution. The brains were removed from the skull and the posterior portion was blocked, and placed in a postfix solution of 4% buffered paraformaldehyde plus 30% sucrose for 2–3 h. The brains were then placed into a 0.1 M phosphate buffer (PB) solution with 30% sucrose for 2 days until they were sunk. Frozen 40 μm thick semi-tangential sections were cut using a sliding microtome. The sections were separated into four numbered series. The first and the third series were processed to reveal the CTb label using a modified version of the CTb protocol described by Angelucci et al. (1996). Sections from the remaining series were processed for cytochrome oxidase (CO) (Wong-Riley, 1989), Nissl substance, or synaptic zinc following the protocol previously described (Khalil and Levitt, 2013, 2017). Sections stained for CO, Nissl substance, and synaptic zinc reveal areal and laminar boundaries as previously described (Khalil and Levitt, 2013, 2014, 2017; Khalil et al., 2018), and were compared with adjacent CTb stained sections to assign tracer injection site and retrogradely labeled cells to particular areas and layers.

### Antibody Characterization

The antibodies used in this study are listed in Table 1. Both antibodies were validated by the manufacturer as follows. The anti-cholera toxin B subunit is a polyclonal antibody raised in goat. Reactivity to cholera toxin B subunit was confirmed by an immunodiffusion assay. The biotinylated rabbit anti-goat IgG is a polyclonal antibody raised in rabbit. This antibody was purified by affinity chromatography using a goat IgG column, and cross-reactivities that are likely to interfere with specific labeling were removed by solid phase adsorption techniques. Cross-reactivity to various immunoglobulins were analyzed by solid phase immunoassay. Furthermore, the specificity and sensitivity of this antibody were also tested on a panel of tissues.

**TABLE 1:** Antibodies used in this study.

Antibody	Source	Manufacturer	Dilution
Anti-Cholera Toxin B Subunit	Goat polyclonal	List Biological Laboratories, Campbell, CA, United States (Cat# 703)	1:5,000
Biotinylated anti-goat IgG	Rabbit polyclonal	Vector Laboratories, Burlingame, CA, United States (Cat# BA5000)	1:200

### Reconstruction of Label

**FIGURE 1:**
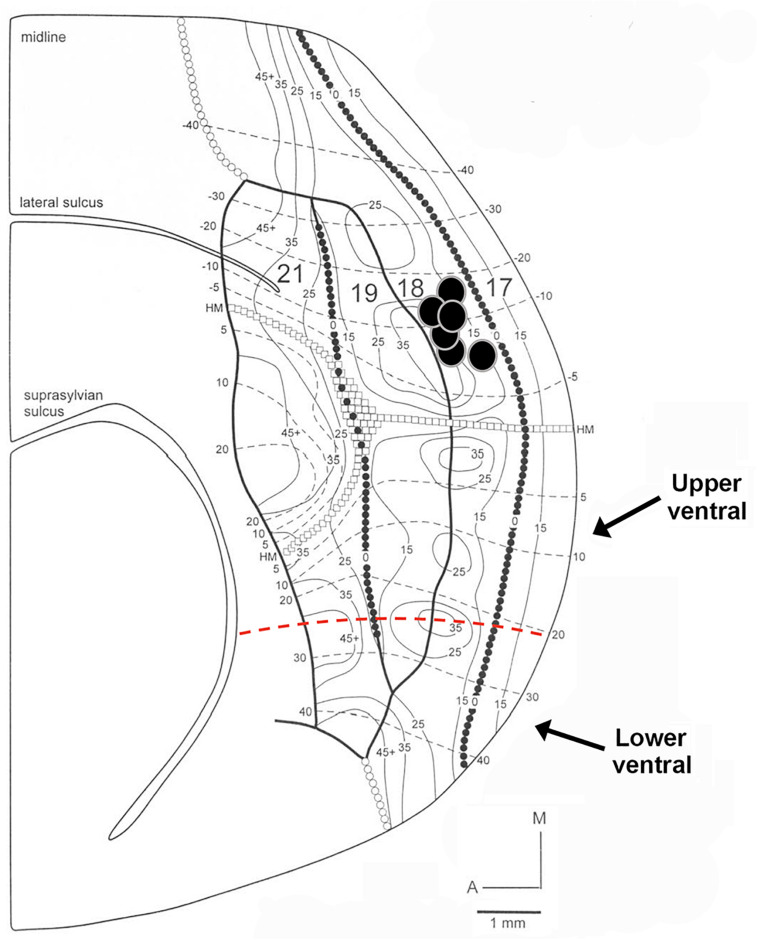
Diagram of retinotopy in areas 17, 18, 19, and 21 in the ferret. The position of each black circle represents the approximate retinotopic location in area 18 of an injection core. Solid and dashed lines represent isoazimuth and isoelevation contours. All injection cores were placed in dorsal cortex representing the lower visual field between 10° and 30° in eccentricity. Two injection cores were closer to the posterior border of area 18 (representing 10°–15° in azimuth), while the remaining four injection cores were closer to the rostral border of area 18 (representing more peripheral azimuths). The horizontal meridian (HM) runs caudo-rostrally and separates dorsal from ventral cortex until it splits in area 21. The dashed red line separates upper ventral from lower ventral cortex. Adapted from Manger et al. (2002). Copyright 2002 by Oxford University Press.

The following criteria were used to ensure that our injections were restricted to area 18, and did not intrude onto area 17 or white matter. The areal and laminar location of the injection core was visually inspected using adjacent sections stained for Nissl substance or synaptic zinc to ensure that none of the cases included in our analysis intruded on area 17. We ensured that our injection cores were sufficiently large in order to label a sizable pool of feedback cells. However, there was slight intrusion onto area 17 in one of our injection cases, so therefore this case was used for comparison. The injection core was defined as the uniform, densely labeled region of CTb. Tracer injections in area 18 were restricted to dorsal cortex representing the lower visual field, with an approximate retinotopic location between 10° and 30° in eccentricity (Manger et al., 2002). The extensive label found in ventral cortex (which does not result after area 17 injections) was interpreted as further evidence that the location of our injections was indeed in area 18. To determine the consistency and strength of ventral label among our area 18 injections, we quantified the proportion of feedback cells in ventral cortex (largely representing the upper visual field) using serial reconstructions. We used maximum dorso-ventral extent of each section to determine the number of feedback cells in the most ventral portion of the section (lower ventral), as well as in the quarter immediately dorsal to that (upper ventral, the dorsal boundary of which approximately coincides with the representation of the horizontal meridian) (see Figure 1). This method was used for all sections to determine the total number of feedback cells found within ventral cortex representing the upper visual field. Analysis of subcortical label provided additional evidence that our injections were indeed restricted to area 18. In agreement with previous reports (Baker et al., 1998; Dell et al., 2019), area 18 injections yielded comparable numbers of retrogradely labeled cells in the C layers as in the A layers (more balanced input). In contrast, if injections intruded onto area 17, there would be many more cells in the A layers of the LGN and a small number of cells in the C-layers.

Section outlines from every fourth semi-tangential section containing CTb label were traced, and retrogradely labeled cells found within each extrastriate visual area (as well as anterogradely labeled cells in primary visual cortex) were plotted in the Neurolucida tracing and reconstruction program (MicroBrightField, Williston, VT, United States). Fiducial marks such as blood vessels and other salient anatomical landmarks were marked, and laminar and areal assignment of CTb labeled cells was then accomplished by comparing CTb tracings with adjacent CO, synaptic zinc, or Nissl stained sections. Assignment of retrogradely labeled cells to specific laminae in tangential sections was accomplished using known laminar variations of different comparison markers. For example, layer 4 in tangential sections was identifiable by the dark CO staining in areas 17 and 18 (for details see Khalil and Levitt, 2013). Because histochemical stains did not always reveal sharp borders between areas (due to the plane of section or intensity of stain), cells found at the borders were assigned to areal boundary zones 18/19 and 19/21. These cell populations constituted on average 3.5% of the total pool of feedback cells and were thus included as separate categories. Fiducial marks were also used to generate three-dimensional reconstructions of section outlines containing CTb label by carefully stacking and aligning tracings of serial sections containing retrogradely labeled cells. Sections containing retrogradely labeled CTb cells were examined and photographed with bright field illumination using a Zeiss Axioimager brightfield microscope. Photomicrographs were enhanced by adjusting contrast and brightness, as well as removing artifacts in image processing software (Adobe Photoshop CS5, v.12). All figures were assembled in Adobe Photoshop (CS5, v.12) and all line graphs and histograms were generated in GraphPad Prism.

### Cell Counts and Cell Densities

To quantify the strength of feedback from extrastriate visual areas to area 18 we determined the proportion of labeled feedback cells in each area. We also determined the proportion of intrinsic cell label in area 18, and feedforward input from area 17. We did not use the absolute number of cells as injection core size and laminar intrusion varied to some extent among cases. We calculated the relative proportion of labeled feedback cells located in each visual area by dividing the number of cells in a given visual area by the total number of labeled cells in extrastriate cortex. Additionally, the proportion of labeled cells located in the different layers of each area was determined by dividing the number of labeled cells in the supragranular, infragranular layers, or layer IV of a given area by the total number of labeled cells found within that area. In each extrastriate area, we determined the peak density of feedback cells as well as the nearest neighbor distance (NND) of each labeled cell using methods previously described in Khalil and Levitt (2014). Briefly, the area containing the highest peak density of labeled cells was delineated with a 200 μm diameter circular region and the volume of each sample was subsequently determined by multiplying the area of the region by the section thickness (40 μm). Sample volumes were then used to yield peak density values. Similarly, NND analysis was performed to assess the spatial distribution of feedback cells in each extrastriate visual area. Specifically, the distribution of NNDs can reveal an underlying regularity in the spatial layout of feedback cells in different visual areas, i.e., it whether the spatial layout of feedback cells is clustered, random, or dispersed regularly as in a lattice. The NND is the distance of a cell to that of the closest adjacent cell in a 2D plane within a given visual area. We determined NNDs within a 300 μm diameter circular region centered over a cluster of feedback cells to include both the densest region of label used to obtain peak density measurements as well as cells in the periphery. We separately computed NNDs in the supra- and infragranular layers in each area and constructed frequency histograms of these values. PD and NND are complementary measures; PD is measured over a small region, whereas NND is measured over a larger portion of the labeled field.

We also assessed differences in geniculocortical inputs to areas 18 and 17 injection cases. This was accomplished by marking and assigning all retrogradely labeled cells in the LGN resulting from area 18 injections to layers A, A1, or C. Similar measures were obtained from two area 17 injections and used for comparison. We subsequently determined cell soma area of retrogradely labeled LGN cells in layers A, A1, and C in area 18 injections and compared with values obtained from two area 17 injections. Although we did not fully reconstruct cell somata, cell soma area was computed as the area of the cell body delineated by drawing a contour around the largest extent of the cell body in a single section.

Statistical analyses were performed in MATLAB (The Mathworks, Natick, MA, United States). Given that our datasets were non-normally distributed we used non-parametric Kruskal–Wallis tests to assess statistical significance among areas and layers with a significance level of *P* < 0.01. *Post hoc* comparisons were then computed using a Bonferroni correction when significance was observed.

## Results

### Description of the Injection Sites

The goals of the present study were to quantitatively characterize the anatomical organization of the feedback pool in extrastriate visual areas that project to ferret area 18, and to determine if feedback projections to area 18 share similar features as feedback projections to area 17 as previously described (Cantone et al., 2005; Dell et al., 2019). By analogy with other studies, we refer to projections that arise from higher order visual areas and terminate in area 18 as feedback projections. We injected the bidirectional tracer Cholera toxin B subunit (CTb) into area 18 of 6 adult female ferrets, which resulted in retrogradely labeled feedback cells in extrastriate cortex. We then determined the complete pattern and distribution of retrogradely labeled cells in extrastriate cortex. Two additional area 17 injection cases were used to examine label patterns in the LGN. All injection cases are summarized in Tables 2, 3. We quantitatively assessed the injection cores for all cases to confirm that the injection was confined to the desired area and to assess how the label characteristics will be influenced by injection core parameters. This method of injection typically yields injection cores with diameters ranging from 912 to 1551 μm. The mean core volume for all six of the area 18 injections was 1.82 mm^3^. The size of the injection core was comparable among four of the cases with the core volume ranging from 2.01 to 2.65 mm^3^, and was substantially smaller for 2 of the cases which had a core volume ranging from 0.84 to 1.18 mm^3^. Our injections were more extensive in the mediolateral direction than in the dorsoventral direction. We determined the total number of labeled feedback cells in extrastriate cortex as a function of injection core total volume. We find a moderate linear correlation between total number of feedback cells and injection core total volume (*r*^2^ = 0.76). However, when we separately plotted number of feedback cells in each visual area as a function of injection core volume we did not observe a linear correlation. This could be due to differences in injection site location, for example potentially injecting monocular zones known to exist in the posterior region of area 18 (White et al., 1999; Manger et al., 2002), which could lead to different connectivity profiles. In most injection cases there was more uptake in the supragranular layers, while in some cases there was equal uptake in supragranular and infragranular layers.

**TABLE 2:** Injection core characteristics for all area 18 cases (Asterisk indicates slight intrusion of injection core onto area 17).

	Case	Core diameter (μm)	Core volume (mm^3^)	DV extent (μm)	ML extent (μm)	Laminar intrusion	Total # labeled cells	Total # feedback cells
**Area 18 injections**	212	1123	2.57	3591	4480	Equal uptake SG and IG	1018	663
	221	912	0.84	1427	2560	Mostly SG/some IG	3915	761
	253	1551	2.01	3194	2560	Mostly SG/little IG	7883	2784
	254	1361	2.65	2258	5120	More SG/some IG	9100	3366
	181	989	1.18	1470	1920	Mostly SG/little IG	2923	515
	217*	1380	2.32	2711	3840	Equal uptake SG and IG	10186	4988

**TABLE 3:** The areal and laminar proportion of labeled feedback cells in all injection cases.

		Area 19	Area 21	Ssy	LT	PP	17/18 border	18/19 border	19/21 border
**212**	Total # cells	233	81	320	0	12	0	9	8
	supra/infra	0.30/0.61	0.32/0.60	0.29/0.65	–	0.25/0.75	–	–	–
**221**	Total # cells	495	64	113	0	14	48	21	6
	Supra/infra	0.29/0.59	0.17/0.77	0.42/0.51	–	0/1.0	–	–	–
**253**	Total # cells	1430	245	907	33	68	0	73	28
	Supra/infra	0.38/0.50	0.17/0.77	0.33/0.43	0.27/0.73	0.10/0.90	–	–	–
**254**	Total # cells	1971	365	810	57	163	0	0	0
	Supra/infra	0.23/0.61	0/0.68	0.07/0.70	0.02/0.98	0.03/0.94	–	–	–
**181**	Total # cells	352	95	44	0	0	12	12	0
	Supra/infra	0.41/0.59	0.04/0.96	0.48/0.52	–	–	–	–	–
**217**	Total # cells	2488	842	1174	86	198	0	106	94
	Supra/infra	0.44/0.38	0.24/0.71	0.33/0.56	0.09/0.91	0.05/0.82	–	–	–

### Spatial Distribution of Label in the Cortex

**FIGURE 2:**
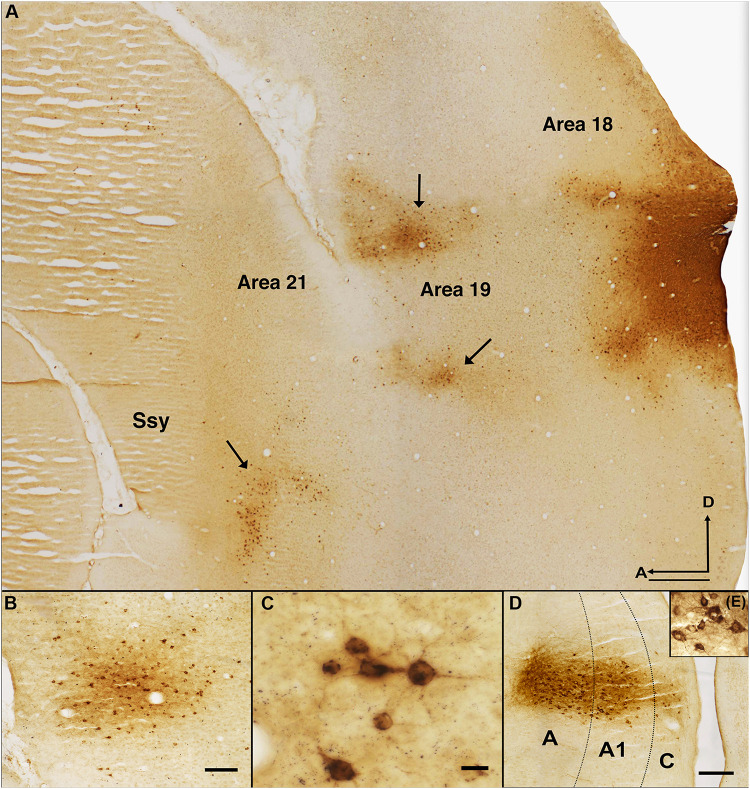
Pattern of feedback label in extrastriate cortex resulting from an area 18 CTb injection. **(A)** Photomicrograph of a semitangential brain section immunoreacted for CTb. Arrows indicate clusters of retrogradely labeled feedback cells. **(B)** Cluster of feedback cells in area 19 of a different section from the same case. **(C)** Higher magnification image of retrogradely labeled cells in area 21 of the same CTb labeled section depicted in B. **(D)** Thalamic label in all the layers of the LGN. **(E)** Higher magnification image of the labeled cells in the LGN. Dotted black lines in **(B)** represent approximate borders between laminae in LGN. Ssy, Suprasylvian cortex; A, anterior; D, dorsal. Scale bar in **(A)** = 500 μm, in **(B)** = 100 μm, in **(C)** = 20 μm, in **(D)** = 200 μm.

Figure 2 reveals the typical pattern of feedback label in extrastriate cortex after a CTb injection in dorsal area 18 of an adult ferret. A representative photomicrograph of a semi tangential section is shown in Figure 2A. The labels indicate visual areas that provide substantial feedback to area 18. Qualitative observations revealed strong feedback connections from the immediately rostral area 19 (black arrowheads) and Ssy (black arrows), with less intense and sparser label found in area 21. We also observed substantially weaker feedback connections from the posterior parietal and lateral temporal areas, and a substantial number of labeled feedforward cells in area 17 (not shown in this figure). Figure 2B shows a cluster of feedback cells in area 19 from a different section of the same case. Figure 2C shows a high magnification image of retrogradely labeled cells in area 21. Extensive thalamic label in the lateral geniculate nucleus (LGN) is revealed in Figure 2D,E is a higher magnification image of labeled cells in the LGN. We did not observe any label in auditory cortex rostral to the Ssy sulcus. This likely reflects our injections not intruding onto sufficiently peripheral representations (Falchier et al., 2002; Majka et al., 2019).

### Projections From the LGN

Prior reports have shown a different pattern of thalamic label in the ferret resulting from area 17 versus area 18 injections. Injections in area 17 result in many labeled LGN cells in the A layers, while few cells are found in the C layer. Conversely, injections in area 18 return comparable numbers of cells in the A and C layers (Baker et al., 1998; Dell et al., 2019). Therefore, to confirm injection locations in area 18 rather than 17, we also compared the absolute number of retrogradely labeled cells in layers A, A1 and C of the LGN.

**FIGURE 3:**
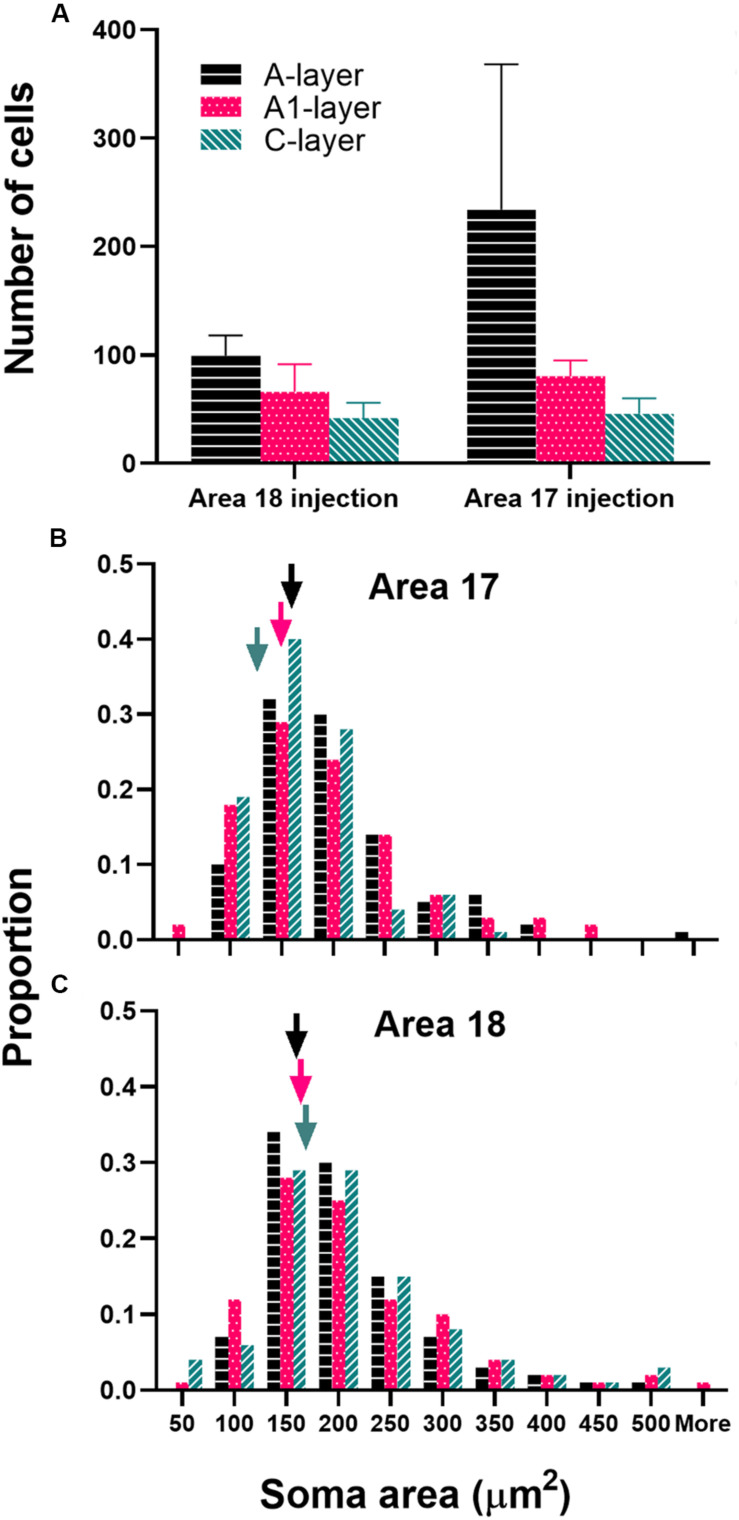
**(A)** Mean number of labeled cells and **(B,C)** soma area in different layers of the LGN resulting from tracer injections into areas 17 and 18. Arrowheads indicate median values in all LGN layers. Error bars represent (+SEM). Black bars denote A-layer, fuchsia bars denote A1-layer, and teal bars denote C-layer.

Our data are consistent with those reported by Baker et al. (1998) in revealing different geniculocortical inputs to area 18 than to area 17. Injections in area 18 resulted in similar numbers of labeled cells in the C layers as in the A layers (more balanced input). The mean number of labeled cells was highest in the A layer of the LGN, followed by A1, and C layers (A-mean = 99, A1-mean = 66, C-mean = 42). This indicates that the majority of the LGN projection to area 18 arises from the A layers (Figure 3A). In contrast, injections in area 17 yielded many more labeled cells in the A layers than in the C layers. The number of labeled cells was highest in the A layer of the LGN, followed by A1, and C layers (A-mean = 234, A1-mean = 80, C-mean = 46). We also measured soma area of thalamocortical relay cells in different LGN layers. The distribution of cell soma area of retrogradely labeled cells found in different LGN layers resulting from area 17 and area 18 injections is illustrated in Figures 3B,C. The arrowheads indicate the median values. The shape of the distribution is similar in all LGN layers and is positively skewed with a long tail. The median cell soma area in all LGN layers resulting from area 18 injections was comparable and did not differ significantly (A-layer = 159.9 μm^2^, A1-layer = 162.8 μm^2^, C-layer = 166.1 μm^2^; Kruskal–Wallis, *P* = 0.765). Similarly, we find a positively skewed distribution of cell soma area resulting from area 17 injections, albeit with a more peaked distribution and less prominent tail (Figure 3B).

We also find that the median cell soma area in the C layer is significantly smaller than in the A1 and A layers when injections were placed in area 17 (A-layer = 159.8 μm^2^, A1-layer = 152 μm^2^, C-layer = 132.1 μm^2^; Kruskal–Wallis, *P* = 0.001). Comparing the median cell soma area in different layers of the LGN between area 17 and area 18 injections we find that the median cell soma area in the C layers were significantly larger in area 18 injection cases (Kruskal–Wallis, *P* < 0.001) compared to area 17 injection cases. Collectively, our LGN data confirm our area 18 injection locations, and suggest different geniculocortical inputs to these cortical areas.

### Comparison With Feedback Projections to Area 17

**FIGURE 4:**
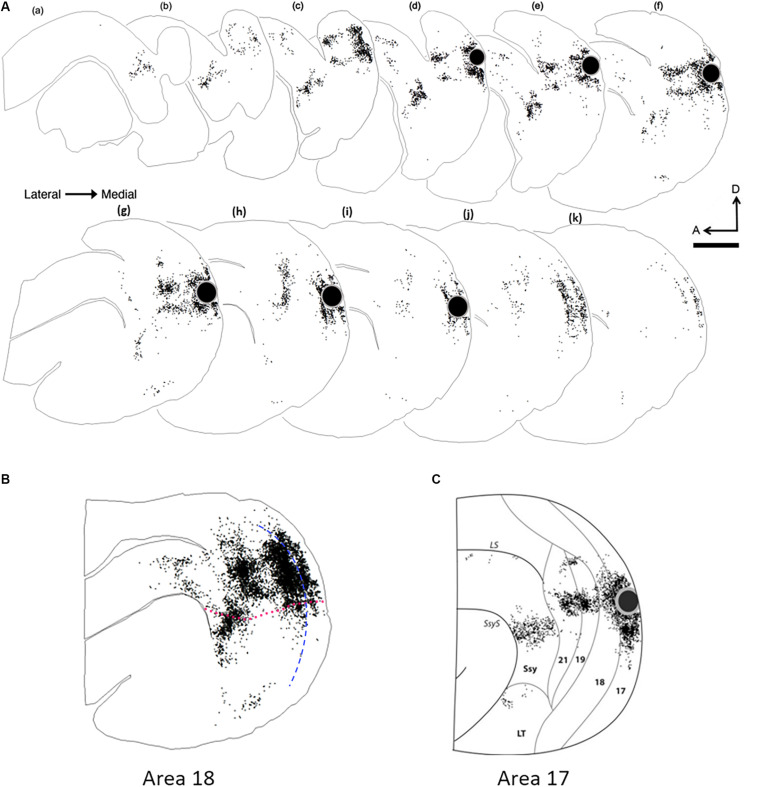
Serial reconstruction of retrograde labeled cells in the visual cortex of an adult ferret resulting from an area 18 injection. The semitangential sections are arranged serially (lateral = left). Black dots represent retrogradely labeled cells. Black circle represents injection core. **(A)** Feedback label in every fourth section in an adult ferret. **(B)** Superimposed and aligned images of the serial sections from the reconstruction shown in **(A)** to reveal the complete pattern of label. **(C)** Collapsed image of serial sections with feedback label resulting from an area 17 injection (Cantone et al., 2005). The sparse labeling shown in **(B)**, anterior to 21 and dorsal to the suprasylvian area (Ssy) is located in areas PPr and PPc, while the labeling that is ventral to Ssy is located in the lateral temporal areas. Dotted fuchsia line in **(B)** separates dorsal and ventral cortex, and represents the approximate location of the horizontal meridian. Dashed blue line in **(B)** represents the approximate areal boundary between areas 17 and 18. LS, lateral sulcus; LT, lateral temporal area. A, anterior; D, dorsal. Scale bar 2 mm in all panels.

To compare the overall pattern of feedback label in extrastriate cortex resulting from area 18 injections with that of area 17 injections, we generated a representative serial reconstruction of label pattern resulting from an area 18 injection case (Figure 4A). We accomplished this by outlining the contour of every fourth section and plotting every labeled feedback cell in extrastriate cortex. Each dot represents a single feedback cell. Superficial sections are to the left with successive sections being more medial. The black circle represents the injection core. Figure 4B shows a collapsed image of the overall pattern that was generated from the precise alignment of all the serial sections. The overall pattern of label resulting from an area 18 injection (Figure 4B) is clearly different from the pattern that an area 17 injection yields (Figure 4C, Cantone et al., 2005). Area 18 injections yield more label in area 19 and Ssy, as well as in the posterior parietal and lateral temporal areas. We delineated dorsal from ventral cortex by first determining the maximum dorso-ventral extent of each serial section, determining the location halfway between, and subsequently drawing a contour in the rostro-caudal direction to delineate dorsal from ventral cortex by linking these midpoints. Thus, the dotted red line in Figure 4B represents an approximate location of the horizontal meridian separating upper and lower visual field representation. Labeled feedback cells in extrastriate cortex were found primarily in the infragranular layers, with a lesser contribution from the supragranular layers, and a minor feedback contribution from layer 4. Feedback labeled cells were often found in dense clusters in areas 19 and Ssy, but in area 21 feedback label was sparser. Labeled feedforward cells in area 17 were found approximately in equal proportions in the supragranular and infragranular layers, with a minor contribution from layer 4.

### Cortical Projections From Ventral Cortex

In the majority of cases, tracer injections in dorsal area 18 resulted in intra-areal and interareal feedback label mainly in dorsal cortex as expected (linking regions with lower visual field representations), but we were also surprised to find significant label in ventral cortex, which represents upper visual fields. We aimed injections away from the area 18 caudal border with area 17, so our injection sites could have intruded onto eccentricities of 20° or beyond (Figure 1). Since isoazimuth lines of eccentricities beyond 20° in the retinotopic map in area 18 (and the mirror image representation in area 19) can form an island and bridge pattern, multiple foci could be labeled in each area, indicating links between matched retinotopic locations. The presence of label in ventral cortex in areas 18 and 19 suggests links between cortical sites of similar eccentricity but in different (upper vs. lower) hemifields. In Ssy, feedback label was also found both in dorsal and ventral cortex at corresponding retinotopic locations [consistent with Cantone et al. (2006) who reported lower visual field representations in Ssy both dorsal and ventral to an upper visual field presentation in this area]. However, due to the area 21 visual field representation, labeled feedback cells in area 21 were largely restricted to dorsal cortex at appropriate retinotopic locations. Two of our injection cases resulted in a pattern of feedback label consistent with injecting the monocular zone in the posterior border of area 18 (White et al., 1999; Manger et al., 2002) as feedback label was largely restricted to dorsal cortex with little feedback label found in ventral cortex. This is presumably because the retinotopic location of these injections was approximately at 10° eccentricity. Another consequence of injecting into the monocular zone is potentially resulting in different connectivity profiles, although we cannot be certain about the nature or extent of these differences. Thus, injections placed closer to the rostral border of area 18 representing the visual field periphery result in intra- and interareal connections in ventral cortex.

**FIGURE 5:**
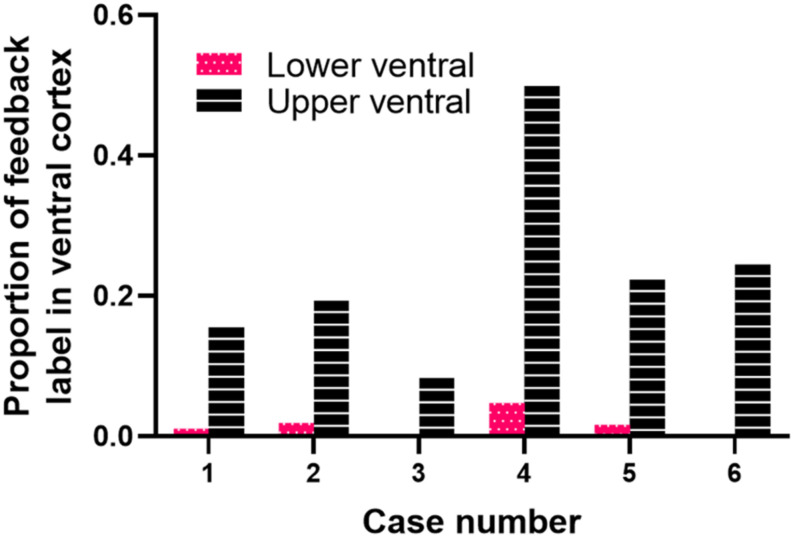
Cortical feedback arising from ventral cortex. The proportion of feedback label in the lower (fuchsia bars) and upper (black bars) halves of ventral cortex.

We quantified the amount of retrogradely labeled cells in ventral cortex resulting from area 18 injections. Figure 5 shows the proportion of feedback cells found within ventral cortex for each case; we divided ventral cortex into upper and lower portions (indicated by arrows in Figure 1, and representing receptive field elevations close or far from the horizontal meridian representation). Fuchsia and black bars represent the proportion of feedback cells in the lower and upper ventral quarters of cortex, respectively. The dorsal portion of ventral cortex contained the majority of feedback label (Range = 9–49%, Mean = 23%), with less feedback from the lower portion of ventral cortex (Range = 0.3–4.7%, Mean = 1.6%). The total proportion of feedback cells in ventral cortex was on average 25% of the total feedback pool. For comparison, we used the same method to determine the proportion of feedback label in ventral cortex resulting from area 17 injections used in a prior study (Khalil and Levitt, 2014). We found that the total proportion of feedback cells in ventral cortex after area 17 injections was substantially smaller (Range = 2–22%, Mean = 11%). Thus, extensive feedback projections from ventral cortex distinguish area 18 from area 17.

### Quantification of Areal and Laminar Distribution of Feedback Cells

**FIGURE 6:**
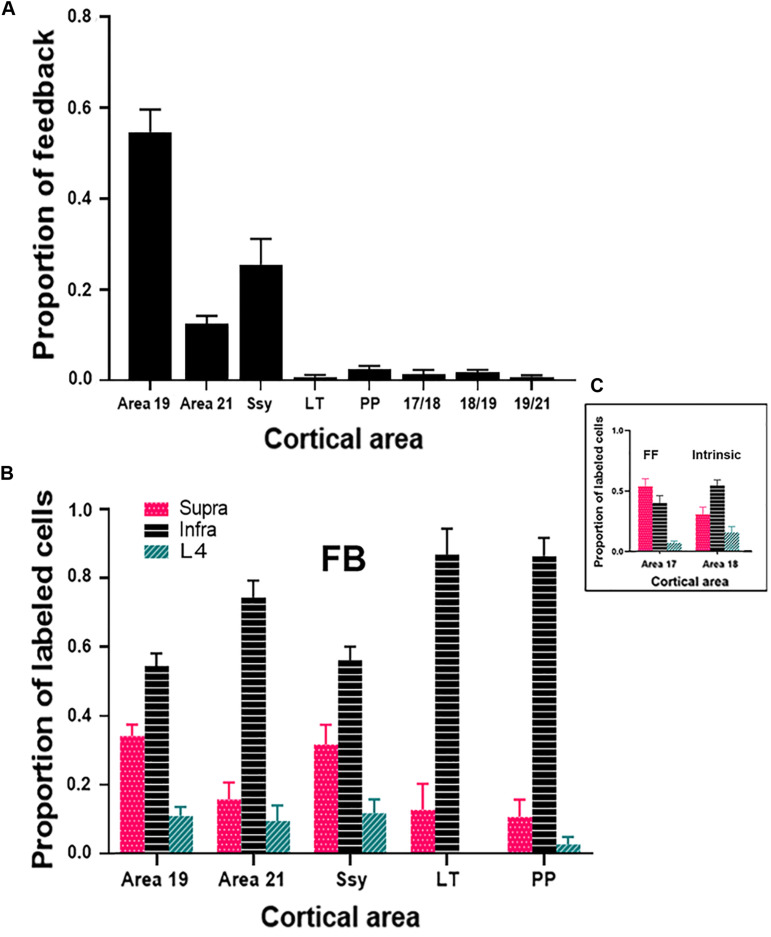
Cortical feedback arising from different extrastriate areas. **(A)** The proportion of feedback projections arising from different visual areas. **(B)** The proportion of feedback arising from the supragranular and infragranular layers, as well as from layer IV. **(C)** The laminar proportion of labeled feedforward-projecting cells in area 17, and of labeled intrinsically projecting cells in area 18. Error bars represent (+SEM). Fuchsia bars denote supragranular layers, teal bars denote layer IV, and black bars denote infragranular layers in both panels **(B,C)**.

To assess the strength of feedback connections from each extrastriate visual area to area 18, we compared the proportion of feedback cells labeled within each area. Previous descriptions characterizing the feedback pool in extrastriate cortex resulting from CTb injections in area 17 of adult ferrets have shown that area 18 provides the greatest contribution of feedback to area 17 (Mean = 45.5%) (Cantone et al., 2005), while areas 19, 21, and Ssy each provide a smaller feedback contribution. We were therefore interested if the pattern of feedback resulting from area 18 injections is similar. Given that the absolute number of labeled feedback cells could vary with injection core size and laminar intrusion, we report the proportion of feedback from each area as a normalized measure that better reflects the strength of feedback arising from each extrastriate visual area. Figure 6A shows the relative proportion of feedback connections arising from each extrastriate area. The proportion of total feedback arising from each visual area differs significantly (Kruskal–Wallis, *P* < 0.001). The greatest proportion of feedback arises from the area immediately rostral to area 18: area 19 (Mean = 55%), followed by Ssy (Mean = 25%), and then area 21 (Mean = 13%). We also observe less prominent feedback to area 18 arising from the posterior parietal (PP) (Mean = 2.5%), as well as the lateral temporal areas (LT) (Mean = 1%). The total proportion of feedback found at areal borders comprises 3.5% of the feedback pool. Extensive intrinsic label was also observed within area 18 as well as feedforward label from area 17. Thus, it appears that similar to area 17, the rostral area immediately adjacent to area 18 provides the greatest proportion of the total feedback pool (area 19).

We next assessed the laminar contribution of feedback projections from each visual area. We report the proportion of labeled feedback cells observed in the supragranular and infragranular layers, as well as in layer IV (Figure 6B). Within each area, the proportion of feedback cells arising from the infragranular layers is significantly larger than from the supragranular layers and layer IV (Infra mean = 72%; Supra mean = 21%; Layer IV mean = 7%, Kruskal–Wallis, *P* < 0.001). This pattern is observed for all extrastriate visual areas (19, 21, Ssy, LT, PP). Figure 6C depicts the laminar proportion of feedforward labeled cells in area 17, as well as the laminar proportion of labeled intrinsic cells in area 18. The majority of feedforward projecting cells in area 17 arise from the supragranular layers, followed by the infragranular layers and then layer IV (Supra mean = 54%; Infra mean = 40%; Layer IV mean = 6%). Intrinsic label found in area 18 had a similar laminar pattern to that observed in feedback projections, with the highest proportion of label found in the infragranular layers followed by the supragranular layers, and then in layer IV (Infra mean = 54%; Supra mean = 31%; Layer IV mean = 15%). Therefore, similar to area 17 injections, within each area there is a greater proportion of feedback arising from the infragranular layers.

### Quantification of Spacing of Feedback Cells

**FIGURE 7:**
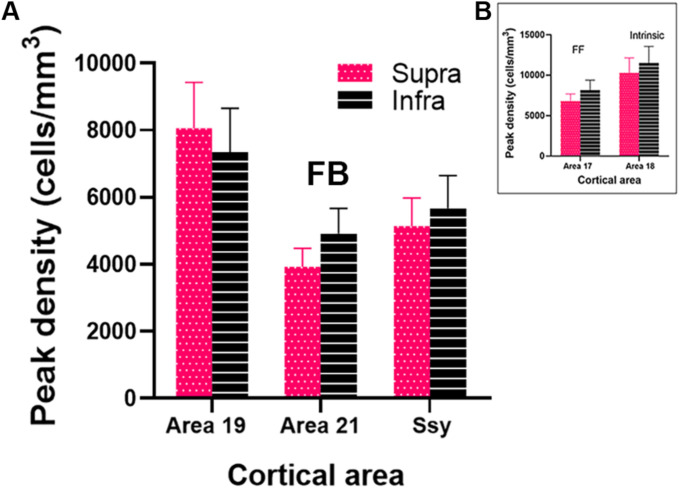
Peak density of labeled cells within the extrastriate visual areas providing feedback to area 18. **(A)** Peak density of labeled cells in areas 19, 21 and Ssy. **(B)** Peak density of labeled cells in areas 17 and 18. Error bars represent (+SEM). Peak density was not computed in areas PP and LT as cell label was sparse. Fuchsia bars denote supragranular layers, and black bars denote infragranular layers in both panels **(A,B)**.

To assess the magnitude and spatial distribution of feedback cells we calculated the peak density of retrogradely labeled feedback cells in areas 19, 21 and Ssy. Peak density is defined as the region of highest cell density within a visual area, delineated with a 200 μm diameter circular region. We separately determined peak density values in the supragranular and infragranular layers in each visual area to reveal potential differences (Figure 7A). Within each area, peak density values did not differ significantly between the supragranular and infragranular layers (Area 19, *P* = 0.539; Area 21, *P* = 0.043, Ssy, *P* = 0.456). Peak density values were greatest in area 19 (Supragranular = 8.06^∗^10^3^ cells/mm^3^; Infragranular = 7.35^∗^10^3^ cells/mm^3^), followed by Ssy (Supragranular = 5.14^∗^10^3^ cells/mm^3^; Infragranular = 5.67^∗^10^3^ cells/mm^3^), and then area 21 (Supragranular = 3.93^∗^10^3^ cells/mm^3^; Infragranular = 4.92^∗^10^3^ cells/mm^3^). Peak density values in both the supragranular and infragranular layers differed significantly among areas (Kruskal–Wallis, infra and supra, *P* < 0.001). We also determined the peak density of labeled cells arising from feedforward connections in the supragranular and infragranular layers of area 17 (Supragranular = 6.81^∗^10^3^ cells/mm^3^; Infragranular = 8.16^∗^10^3^ cells/mm^3^, Figure 7B). The high peak density values of feedback cells in area 19 are comparable to those of feedforward labeled cells in area 17. Likewise, the peak density of feedforward labeled cells in area 17 is only 28% greater than feedback peak density in Ssy and 41% greater than feedback peak density in area 21. Lastly, the peak density of intrinsic cells in area 18 is 10.03^∗^10^3^ cells/mm^3^ in the supragranular and 11.51^∗^10^3^ cells/mm^3^ in the infragranular layers (Figure 7B). These data highlight the significance of FB cortical input from multiple visual areas to area 18. Furthermore, area 18 injections result in similar peak density values of FB cells between the supragranular and infragranular layers, whereas area 17 injections lead to higher peak density values of FB in the infragranular layers.

**FIGURE 8:**
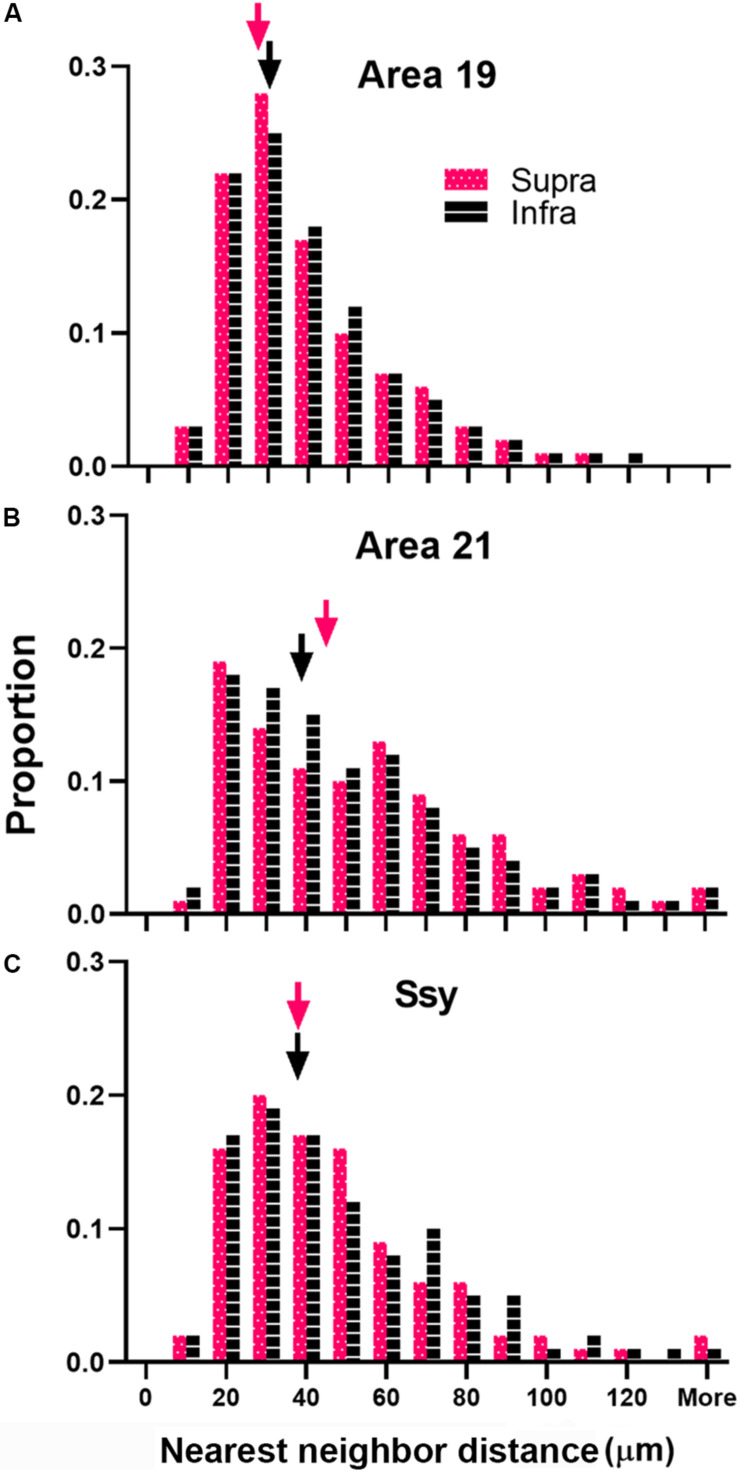
Distribution of nearest neighbor distances (NNDs) between feedback cells in area 19, 21, and Ssy. Frequency histogram of NNDs in the supragranular and infragranular layers of **(A)** Area 19, **(B)** Area 21, and **(C)** Ssy. Arrowheads indicate median values. Fuchsia bars denote supragranular layers, and black bars denote infragranular layers.

We next considered the spatial distribution of cells providing feedback to area 18 in area 19, 21, and Ssy by assessing the NND between labeled cells in each visual area. We separately calculated the NND values in the supragranular and infragranular layers of areas 19, 21, and Ssy. The NND value reflects the distance between every cell within our region of interest and its closest neighbor. While our peak density measures were obtained from circular regions with a 200-μm diameter, our NND values were similarly obtained from the same region but encompassed more of the feedback cluster (300 μm diameter). Figure 8 shows the distribution of NND values between feedback cells in the supragranular and infragranular layers in areas 19, 21, and Ssy respectively. Higher median NND values indicate that labeled cells are more sparsely distributed. The fuchsia and black arrowheads indicate the median NND values in the supragranular and infragranular layers respectively. Although the shape of the distribution for all areas is positively skewed with a prominent tail, the shape of the distribution in area 19 is more peaked than in areas 21, and Ssy with a lower median NND value in the supragranular and infragranular layers (Supra NND = 28.8 μm; Infra NND = 30.3 μm). This reflects the higher density of feedback label in area 19. We find a more prominent tail in the distribution of NNDs in areas 21 and Ssy leading to higher median values in the supragranular (Median NND in area 21 = 45.3 μm; Median NND in Ssy = 37.1 μm), and infragranular layers (Median NND in area 21 = 38.7 μm; Median NND in Ssy = 37.1 μm). Furthermore, the distribution of NNDs in areas 19 and Ssy appear similar suggesting a common spatial arrangement of feedback cells.

**FIGURE 9:**
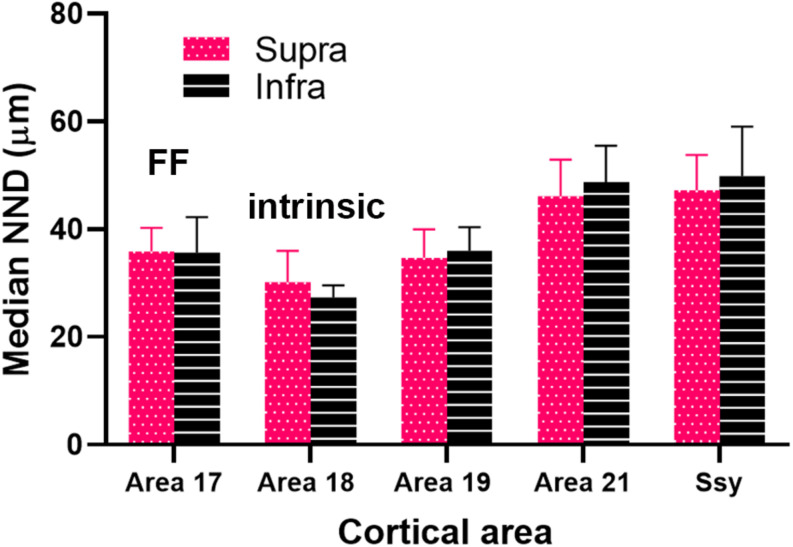
Median nearest neighbor distance (NND) between feedback cells in the supragranular and infragranular layers of each area. Error bars represent (+SEM). Fuchsia bars denote supragranular layers, and black bars denote infragranular layers.

We plotted the median NND value in the supragranular and infragranular layers of all visual areas providing feedback to area 18, as well as in area 17 (feedforward projections) and area 18 (intrinsic connections) (Figure 9). The NND values in the supragranular and infragranular layers in each visual area did not differ significantly from one another (Kruskal–Wallis, *P* < 0.765). However, the median NND value in the supragranular layers of all areas providing feedback to area 18 differed significantly among areas (Kruskal–Wallis, *P* < 0.0001). Similarly, the median NND value in the infragranular layers of all area providing feedback to area 18 differed significantly among areas (Kruskal–Wallis, *P* < 0.0001). Furthermore, our NND values in all areas examined are consistent with peak density values reported in Figure 7. For example, the higher peak density values of feedback cells observed in area 19 are mirrored by lower NND values. These data suggest that within each area, the NND values of feedback cells are similar in the supra- and infragranular layers, and that FB cells are more closely spaced in the area immediately adjacent to area 18.

## Discussion

We found several similarities to the FB projection to area 17. First, multiple visual cortical areas provide feedback to area 18: areas 19, 21, Ssy, and weaker inputs from posterior parietal and lateral temporal visual areas, largely linking retinotopically corresponding regions. Feedback projections arising from the infragranular layers was approximately threefold higher than from the supragranular layers. Also, the cortical area immediately rostral to area 18 provides the greatest proportion of total cortical feedback, and has the greatest peak density of cells providing feedback to area 18. The spacing of cells in extrastriate cortex providing feedback to areas 17 and 18 are similar. Our data are also consistent with those of Baker et al. (1998) in suggesting different geniculocortical inputs to area 18 than to area 17. We also found several differences in the organization of feedback circuits that project to areas 17 and 18. Unlike area 17, there is substantial input to dorsal area 18 (representing lower visual fields) from ventral cortex (representing upper visual fields). Furthermore, area 17 injections result in a greater proportion of FB from area 21 than from Ssy, whereas area 18 injections result in more FB from Ssy than from area 21.

### Comparison With Feedback Circuits to Area 17 in the Ferret, Cat, and Primate

In general, area 18 injections in adult ferrets resulted in retrogradely labeled feedback cells in the same cortical areas that send feedback projections to area 17 (19, 21, Ssy, PP, and LT) (Cantone et al., 2005; Dell et al., 2019), albeit with different proportions. Furthermore, it appears that area 19 provides the greatest feedback contribution, followed by Ssy and then area 21. This finding is in line with previous observations in ferrets (Cantone et al., 2005; Dell et al., 2019), and primates (Kennedy and Bullier, 1985; Perkel et al., 1986; Barone et al., 1995) which reveal that the area immediately rostral to a given area supplies the majority of feedback. We did not observe any label in auditory cortex rostral to the Ssy sulcus as our injections may not have been peripheral enough (Falchier et al., 2002; Majka et al., 2019). We did not systematically investigate connections at different eccentricities so we cannot compare to other studies (Palmer and Rosa, 2006). The observed differences in the proportion of feedback from each of areas 19, 21 and Ssy to area 18 suggests that there may be unique functional roles each circuit plays in modulating the responses of neurons in area 18. Therefore, it appears that areas 17 and 18 are broadly connected via cortical feedback projections from the same extrastriate areas.

In the adult ferret, the majority of feedback cells that target area 18 originate primarily from the infragranular layers, with fewer cells arising from the supragranular layers. Similarly, injections in area 17 of the adult ferret result in a higher proportion of feedback projections from the infragranular than the supragranular layers (Cantone et al., 2005). In the cat, feedback projections from the middle suprasylvian region that target area 18 arise mostly from the infragranular layers (Payne and Lomber, 2003). Furthermore, studies in primates (Kennedy and Bullier, 1985; Felleman and Van Essen, 1991), cats (Batardiere et al., 1998; Barone et al., 2000; Grant and Hilgetag, 2005; Hilgetag and Grant, 2010), and rodents (Coogan and Burkhalter, 1990), also show that the laminar pattern can be informative of hierarchical organization, whereby the proportion of feedback label in the supragranular layers decreases along the visual cortical hierarchy (Markov et al., 2014). Therefore, documenting the laminar pattern of feedback projections to area 18 in the ferret is central to understanding cortical hierarchy and the functional roles of ferret visual cortical areas. In the current study, given the proportion of labeled cells in the supragranular layers is comparable among areas 19, 21 and Ssy, these areas could represent one level in the hierarchy. Similarly, the proportion of labeled cells in the supragranular layers in areas PP and LT is also comparable and suggests that these areas are on a different level in the visual cortical hierarchy.

We further assessed the spatial distribution of retrogradely labeled feedback cells by separately measuring peak density in the infragranular and supragranular layers. Peak density is highest in area 19, followed by Ssy then area 21, which mirrors the pattern observed for the areal proportion of feedback label discussed above. Thus, the peak density values of feedback cells is greatest in the area immediately rostral to area 18 (area 19); this too is similar to findings reported by Cantone et al. (2005), whereby injections in area 17 result in the highest peak density of feedback cells in the area immediately rostral to area 17 (area 18). However, the absolute peak density values reported by Cantone et al. (2005) are lower than the values in the present study. The highest peak density value they report was in area 18 and approximately 3000 cells/mm^3^, whereas the highest value we observed (8000 cells/mm^3^) was in area 19. This may be attributed to differences in injection size as injection core volumes in our study were larger than ones reported by Cantone et al. (2005). We also observed that the peak density values of feedback cells to area 18 from the infragranular and supragranular layers is similar in all areas. In contrast, Cantone et al. (2005) found that the peak density values of feedback cells to area 17 is higher in the infragranular layers than the supragranular layers.

Our nearest-neighbor analysis revealed that median NND values of FB cells in the supra- and infragranular layers were lowest in area 19, followed by area 21 and Ssy. These results confirm our peak density values as peak density of FB cells was greatest in area 19. Furthermore, median NND in the supra- and infragranular layers was similar for all areas, which is also consistent with the peak density results. The distribution of NNDs is informative as it reveals potential differences in the spatial layout of feedback cells in different visual areas. Specifically, it can indicate if the spatial layout of feedback cells is clustered, random, or more dispersed. Although the shape of the distribution of NNDs is positively skewed in all areas, it appears more peaked in area 19. Consequently, approximately 52% of NND values in the supra- and infragranular layers of area 19 are 30 μm or less. This suggests that the majority of FB cells in area 19 are closely spaced, forming clusters, while the remaining FB cells are spaced further apart and are more dispersed. However, the shape of the distribution in area 21 and Ssy is less peaked and is characterized by a prominent tail with longer NND values. This further reflects the presence of a subpopulation of FB cells in area 21 and Ssy that is more sparsely distributed.

An unexpected finding is the similar NNDs of FB cells in area 21 and Ssy, although peak density in and relative proportion from area 21 are lower than in Ssy (suggesting a weaker input to area 18 from area 21). This finding could be accounted for by the different size circular regions used to measure peak density and NND. The slightly lower peak density values in area 21 compared to Ssy yet similar NND values imply that cells are more uniformly distributed in both the smaller and the larger region. Conversely, FB cells in Ssy appear to be closer together (i.e., higher PD) in the smaller region and further apart in the larger region. FB cells clustered together at high density likely have similar receptive field locations. The mismatch between PD and NND values (due to differences in how FB cells are spread across an area) implies differences among different corticocortical inputs to area 18 with regard to retinotopic correspondence between source and target. Collectively, these findings suggest that similar peak density values across visual areas implies similar FB weights, while similar NND values suggest a similar spatial layout. Each measure describes a different attribute of the feedback pool. Multiple peaks in the distribution of NND values would indicate clustering or patchiness of the population of feedback cells; we found no evidence for clustering of cells providing feedback to are 18 from any cortical source. These results are largely in agreement with the pattern of feedback to ferret area 17 at a late (10 weeks) stage of development (Khalil and Levitt, 2014). The authors showed median NND is highest in Ssy, followed by area 21, area 19, and then the immediately rostral area (area 18), with similar values in supra- and infragranular layers. Markov et al. (2014) used the surface area of label as a measure of spatial distribution, reporting that the retrograde label found in V2, V3 and MT after a V1 injection is more widely spread in the infragranular layers than the supragranular layers. They also observed that the surface area is largest in V2, followed by MT and then V3. These results support our findings of feedback connection strength and spatial distribution following an area 18 injection in the adult ferret, whereby area 19 has the highest proportion of label, the highest peak density, and the lowest NND values; this is followed by Ssy and then area 21.

Our data are consistent with those of Baker et al. (1998) in revealing differential geniculocortical inputs to area 18 than to areas 17. Area 17 injections lead to predominantly A-layer label while area 18 injections result in a comparable number of cells in the A- and C-layers. Additionally, cell soma area of the C-layer neurons that project to area 18 appear to be significantly larger than those that project to area 17; this too is in agreement with reported findings by Baker et al. (1998). Differential thalamic input to these cortical areas may also contribute to their distinct functional roles.

### Comparison With Feedback to Area 18 in Ferret, Cat, and Primate

Our results are in agreement with prior studies in primates that reveal feedback connections to V2/area 18 from different source areas and layers (Rockland and Pandya, 1979; Kennedy and Bullier, 1985; Ungerleider et al., 2008; Borra and Rockland, 2011; Nascimento-Silva et al., 2014). However, these studies have largely been qualitative in nature describing the overall pattern of feedback to area V2/area 18. Our findings are largely consistent with findings in cats (Payne and Lomber, 2003) in revealing that feedback from each cortical area arises mostly from the infragranular layers. However, our results reveal that area 19 supplies the greatest feedback contribution to area 18, followed by Ssy, and then area 21. Other studies in the cat have documented prominent feedback connections to area 18 from ipsilateral area 7 (Yang et al., 2016), areas 20/21 (Bullier et al., 1984), and PMLS (Segraves and Innocenti, 1985). Thus, primate and cat studies have largely reported on areal and laminar sources of feedback connections to V2/area 18. Importantly, anatomical reports characterizing the organization of feedback connections to ferret area 18 are scarce. In a recent tract tracing study, Dell et al. (2019) examined the connectivity of FB circuits in ferret visual cortex, primarily reporting qualitative data on the overall pattern of FB projections after injections in area 17, 18, 19, and 21, as well as the fraction of labeled FB neurons.

Our results also show that Ssy provides a greater feedback contribution to area 18 than area 21. This is unlike the pattern found by Dell et al. (2019), whereby the authors report that area 21 provides more feedback to area 18 than does Ssy. However, our finding is consistent with that of Payne and Lomber (2003) which report that feedback from the middle suprasylvian region in the cat (PMLS, AMLS, and PLLS) comprises 26% of all feedback inputs to area 18. Similarly, our findings are also consistent with those of Connolly et al. (2012), who used viral tracers to reveal a substantial feedback contribution from cat PMLS and PLLS to area 18. Furthermore, when different measures of feedback strength are considered in other studies, a similar trend is observed whereby the second visual cortical area rostral to the injected area sends less feedback than the area immediately rostral to it. This was shown by Cantone et al. (2005), who reported that the peak density of labeled feedback cells projecting to area 17 in the ferret is higher in area 21 than in area 19. Thus, the major feedback contribution area 18 receives from Ssy appears to be functionally relevant as discussed below.

Our injections were typically placed in the dorsal region of area 18 resulting in intra- and interareal label in ventral cortex. The presence of ventral label could be due to injection sites in far peripheral representations (Gattas et al., 1997), but we did not systematically explore this possibility so we cannot rule it out. Interareal feedback label which was typically observed in clusters, was found at retinotopically appropriate locations. This finding mirrors that in marmoset visual cortex (Jeffs et al., 2009), whereby the authors report intra- and interareal label in ventral cortex resulting from injections placed near the horizontal meridian at the rostral border of dorsal V2. Thus, given differences in retinotopic maps between areas 17 and 18 we find that while many aspects of feedback projections from extrastriate cortex to areas 17 and 18 are broadly similar, the topography is fundamentally different due to differences in visual field representations.

### Physiological Relevance of Feedback Connections Targeting Areas 17 and 18

The physiological role of feedback connections has been extensively studied in primates and carnivores. In primates, feedback projections arising from higher order areas that terminate in area 17/V1 are thought to underlie contextual effects and thus contribute to global integration of visual signals. For instance, feedback from area V5/MT to areas V1 and V2 in monkeys has been shown to modulate the center-surround responses of neurons (Hupé et al., 1998; Angelucci et al., 2002), thus playing a role in figure-ground segregation (Bullier et al., 2001; Hupé et al., 2001). Feedback from V2 to V1 in primates controls the size of the RF in V1 cells by increasing responses to the RF center and suppressing responses to the RF surround (Nurminen et al., 2018). Furthermore, in cebus monkey, feedback from V4 to V2 modulates direction and orientation selective responses of V2 neurons (Jansen-Amorim et al., 2012). Similarly, in cats, feedback projections from higher order visual areas modulate different response properties of neurons in lower order visual areas. For instance, feedback from area 7 (polysensory association area that responds to visual, somatic, and auditory stimuli) to areas 17 and 18 in the cat has been shown to modulate the spatial frequency of neurons in these areas (Yang et al., 2016). Additionally, feedback from the posterior middle suprasylvian region (pMS) can decrease the response of neurons in cat area 18 to orientation and direction-selective stimuli (Galuske et al., 2002), whereas feedback from cat area 21a modulate the response amplitude of orientation maps in areas 17 and 18 that is spatial frequency dependent (Huang et al., 2004). In cats, studies in which postero-temposal visual (PTV) cortex is reversibly inactivated have also provided evidence for the modulatory effects that FB projections from extrastriate cortex exert on neurons in area 17. Inactivation of ipsilateral PTV by cooling leads to a significant reduction in response magnitude to visual stimuli in the classical receptive field (CRF) of cells in area 17 (Bardy et al., 2006, 2009; Huang et al., 2007). Similarly, upon inactivation of PTV, a significant reduction in the relative strength of extra-classical receptive field (ECRF) modulation of the CRF-induced spike-responses was observed. Findings from a similar study whereby PTV cortex was inactivated has shown that feedback from PTV modulates responses in area 17 and area 19 and depends on stimulus velocity and direction selectivity (Huang et al., 2017). Collectively, these results provide evidence that V2/Area 18 receives inputs from multiple sources, which modulate its activity in different ways, leading to a contextually relevant response.

In the adult cat, the lateral suprasylvian and middle suprasylvian areas (also referred to as suprasylvian gyrus) have been shown to be involved in the visuomotor initiation of saccadic eye movements, the regulation of attention to visual cues, and visual perception through the generation of behavioral space (Joseph and Giroud, 1986; Yin and Greenwood, 1992). Ssy in the ferret is homologous to cat PMLS and is involved in motion and direction processing. Ssy, which is also referred to as PMLS is linked to the parietal cortex, and seems to have a pivotal role in the dorsal processing stream (Cloutman, 2013; Dell et al., 2019). This may explain the strong feedback from Ssy to area 18 we observe in our study, as this feedback circuit could be functionally important for the dorsal processing stream. Recently, Lempel and Nielsen (2019) have shown that PMLS in the ferret plays an important role in the cortical motion-processing pathway, similar to area MT’s function in primates. Furthermore, this dorsal motion-processing cascade has been shown to generate spatial perception and visuomotor performance in primates, with area MT (homologous to Ssy) providing a connection between early visual areas and the parietal lobe (Ungerleider and Desimone, 1986). Our results confirm a strong connection between Ssy and area 18 in the adult ferret, supporting its importance in motion-processing and suggesting that feedback from this higher-order visual area may have a significant modulatory role on neurons in area 18. Neurons in area 18 of the cat respond well to high-speed stimuli, which might also reflect the feedback influence of Ssy/PMLS and area 7 (equivalent to ferret PPc), which has also been shown to send direct feedback to area 18 (Yang et al., 2016).

## Permission to Reuse and Copyright

Permission to reproduce Figure 4A from Cantone et al. (2005) was obtained from the licensed content publisher John Wiley and Sons with license number 4742971370339.

Permission to adapt Figure 9 from Manger et al. (2002) was obtained from the licensed content publisher Oxford University Press with license number 4897490497226.

## Data Availability Statement

The raw data supporting the conclusions of this article will be made available by the authors, without undue reservation.

## Ethics Statement

The animal study was reviewed and approved by the City College of New York Institutional Animal Care and Use Committee.

## Author Contributions

RK, MS, and JL carried out the experimental procedures. RK, MS, SA, and JL performed the data acquisition and analysis. RK, SA, and JL were responsible for writing and revising the manuscript. All the authors contributed to the article and approved the submitted version.

## Conflict of Interest

The authors declare that the research was conducted in the absence of any commercial or financial relationships that could be construed as a potential conflict of interest.
